# *‘I tried to take my phone off my daughter, and i got hit in the face’*: a qualitative study of parents’ challenges with adolescents’ screen use and a toolbox of their tips

**DOI:** 10.1186/s12889-024-17690-z

**Published:** 2024-01-18

**Authors:** Samantha Marsh, Joanna Ting Wai Chu, Amanda Jane Calder

**Affiliations:** 1 School of Population Health, University of Auckland, Auckland, New Zealand; 2 National Institute for Health Innovation, UniServices, Auckland, New Zealand

**Keywords:** Adolescent behavior, Adolescent health, Family health, Screen use behaviour, Technology

## Abstract

**Background:**

Concerns about adolescent screen use are often expressed but poorly understood, particularly in terms of how parents are supposed to respond in ways that balance safety, care and developing independence and autonomy. This qualitative study investigated parental perceptions and concerns about screen use of adolescents aged 13 to 17. Current strategies to manage screen use and barriers to doing so were explored, and parents were asked to outline recommended interventions for better outcomes.

**Methods:**

Interviews and focus groups were held with 33 adults in Auckland, Aotearoa New Zealand (mean age 48 years) who were parenting adolescents (mean age 14 years). Interviews were transcribed verbatim, then inductive analysis and interpretation by the research team distilled the key ideas and illustrative quotes. A table of recommendations for a practical ‘toolbox’ was developed from these in-depth conversations.

**Results:**

Parents reported extensive use of screens by their adolescents, constantly throughout the day (and night, instead of sleeping). Four areas of specific concern included: (1) addict-like behavior, (2) exposure to harmful (and inane) content, (3) living in a virtual world, and (4) negative impacts on physical, mental, and cognitive wellbeing. To manage adolescent screen use, family rules and restrictions (on time and place) were common. Some used technical control via software or accessing the adolescent’s devices and/or accounts to check for inappropriate content (such as pornography). Communication about device use and self-regulation were important. Barriers to managing screen use included trying to avoid conflict with their child; difficulties with consistency or follow-through on rules; lack of technical knowledge; parental screen use that set a bad example; and device use needed for school or other purposes. Recommendations from parents are presented in a toolbox of tips and techniques they shared, and their ‘wish list’ for better access to practical, local, scientific information, examples of techniques that have worked for other families, tools for problematic behavior and risk (including how to begin conversations with adolescents about their concerns), and having schools and young people involved in developing interventions to build digital citizenship.

**Conclusions:**

Rich, nuanced accounts from parents about adolescent screen use in their families and communities underpinned their practical ideas for more skillful responses to young people grappling with an addictive digital existence.

## Background

With the rapid adoption of technological devices, screens have become a ubiquitous part of life. However, excessive screen use has been linked to numerous negative outcomes for adolescents [1], including but not limited to poor academic performance [2], low sleep quality [3], attention problems [4], and decreased psychological well-being [5]. Studies suggests that adolescents are spending up to 11 h per day using screen-based technologies, such as smartphones, tablets, computers, and TV [6, 7]. Recent research advances call for a focus not only on hours on screen, but also to develop a more nuanced understanding of how specific online experiences have harmful effects on young people and family relationships [8], with a recent US Surgeon General’s report highlighting wide-ranging concerns [9]. Finding ways to negotiate digital life with adolescents is undoubtedly important to their health and wellbeing [10] and raises ongoing and challenging questions.

Parents are a key influence on adolescents’ screen use by providing them with screen devices and setting family rules, as well as through their attitudes and own screen use. Numerous studies have shown strong association between parents’ and children’s use of screens [11–14]. There are also many challenges, as screens are the latest focus of what has been described, for many generations, as “the eternal parental tension between protecting adolescents’ safety and nurturing their autonomy” (p. 1687) [15]. 

Much research in this area is quantitative [16, 17] or focused on young children [18, 19]. In-depth understanding of parents’ perceptions and experiences with managing adolescent screen use in the family is needed [7], to provide “context and a human voice” to ongoing debates [19]. Qualitative insights are important for developing effective strategies to support parents on managing adolescent screen use [20].

This paper reports on the formative work conducted with parents on their perception and management of adolescent screen use, as part of a wider project in Aotearoa New Zealand that aims to develop and evaluate the effectiveness of a parenting intervention aimed at promoting healthy screen use in families. Specifically, we were interested in parents’ perceptions and concerns around adolescent screen use, family rules and strategies around screens, challenges and barriers with implementing such strategies, and their recommendations for interventions that could help parents.

## Methods

We aimed to explore the perceptions and concerns of parents in Aotearoa New Zealand around their adolescents’ screen use, using a general inductive qualitative approach to explore their subjective experience, as is suitable to an exploratory initial study of this type [21]. Individual interviews and focus groups were conducted face-to-face with parents of adolescents to explore their perceptions of screen use in their children and to garner a deeper understanding of what parents do—or try to do—about adolescent screen use [22]. Focus groups enable rich discussions from multiple perspectives [22]. A senior social science researcher conducted the interviews and focus groups using a semi-structured discussion guide to allow for flexibility and novel points to be explored [23]. The interview guidelines and prompts were developed by the research team to cover a range of key issues related to the research questions, while also allowing flexibility to explore issues not before canvassed in the literature.

### Participant recruitment

Prospective participants were parents of children aged 13 to 16 years who were invited to take part in the study through advertisements on social media, snowball sampling, and word of mouth. Participant criteria included having concerns about the screen use of their adolescent/s. Researchers spoke to participants to outline the study, check they met criteria, and ask if they wanted to join a focus group or have an individual interview. All participants were provided with an information sheet that outlined the background to the study, the research process, and how the information would be used. Participation was voluntary and a written informed consent agreement was signed prior to taking part in an interview or focus group. The research project and all documents had been given ethical approval by the [blinded for review] ethics committee.

### Data collection

Ten individual interviews and six focus groups were conducted with 33 parents of adolescents (13–17 years). Interviews were approximately 50 min duration, and the focus groups were approximately 90 min, with two to five people in each. Topics explored included parents’ observations of their adolescent’s screen use and their specific concerns about it. They were also asked about their attempts to limit screen use, including what worked, the effects on the young person and what the barriers were to limit-setting. Finally, they were asked about what they would find helpful in an intervention designed to assist parents with managing adolescents’ screen use. Interviews were held in person, at a location of the participant’s choosing (e.g., home, a local café); focus groups were held in a spacious meeting room. Each participant received a shopping voucher in appreciation of their time, and refreshments were provided.

### Data analysis

All interviews and focus groups were audio-recorded and transcribed verbatim. Transcripts were entered into NVivo 10 software (QSR International, Warrington UK) to support management of the dataset when coding and analyzing the texts [24]. A general inductive approach [21] was used to interpret the data in relation to the interview topics. Inductive approaches are intended to aid an understanding of meaning in nuanced conversations through the development of summary themes from raw data, without highly theoretical filters [21]. The coding framework used was developed through close reading of the transcripts; identifying segments of text and creating categories for those segments; reviewing the categories to minimize repetition and redundancy; and distilling key categories that could encompass the main ideas [24]. A number of strategies were employed to ensure research rigor, including research team review of the coding framework, agreeing on the main categories being analyzed, reviewing the final report and, now, summarizing key findings for this article [25].

## Results

Participant demographics are presented in Table 1. Participants were predominantly female (79%), NZ European (66%) or NZ Indigenous Māori (15%), with an average age of 48 years (range 34 to 58 years).

**Table 1:** Participant Demographics

	Number	Percent
***N***	33	-
**Gender**		
Female	26	79
Male	7	21
***Parent Details***		
**Age**	34–58 (*M* = 48 years)	
**Ethnicity**		
NZ European	22	66
Māori	5	15
Other	6	18
***Child Details***		
**Age**	13–17* (*M =* 14 years)	-
**Devices Used**		
Smartphone	26	79
Computer	23	70
Tablet	12	36
Video-game	11	33
Other	2	6

Note: * Study criteria were parents of children aged 13 to 16 years; one child had turned 17 years in the time between recruitment and interview

The adolescents they were talking about had an average age of 14 years; the most widely used device was a smartphone (79%), followed by a computer/laptop (70%), with most adolescents having access to two or more devices. We did not collect information on the child’s gender.

Overall, young people’s screen use was a significant issue for participants, with negative impacts reported for the adolescents, their parents and, in some cases, entire families. Balancing parental management strategies with maintaining a relationship with their adolescent was challenging, and barriers to management were wide-ranging. Participants shared many practical ideas of what might help as a ‘toolbox’ of ideas for other parents and for further community/public health development.

An overview of each topic is discussed, with illustrative verbatim quotes (with filler words “um” etc. removed for readability). There was a lack of marked differences between the interview and focus group responses, or by gender or culture, so we present the data here collectively with no identifiers [26]. (We consider this issue further in the *Discussion*.)

### Parents are concerned about adolescent screen use: ‘***a complete waste of a wonderful mind and body***’

Although some utility of screens was acknowledged, parents perceived screen use overall to be negative. Out of all devices, smartphones were viewed as particularly problematic as they were portable, with adolescents constantly receiving messages and notifications. Parents noted four specific concerns: (1) addict-like behavior, (2) exposure to harmful content, (3) living in a virtual world, and (4) negative impacts on physical, mental and cognitive wellbeing.

#### Addict-like behavior: ‘it is literally an addiction’

Parents often relied on the metaphor of “alcohol and drug addiction” to describe their adolescents’ relationship with screens. They referred to “the cycle”, the concept of “the more you have it, the more you want it”, and negative withdrawal behavior, such as anger, deception, lying and moodiness, “which is what an addict does”. This metaphor appeared particularly pertinent when describing adolescents’ reactions to parental attempts to monitor or reduce screen use:

I think it is literally an addiction… we’d hide all the devices so he couldn’t play on it, he’d look around for them, and it was just mental… I honestly think that if you just left them to it, they’d sit there wallowing in their own feces, and not eating until they died.

Participants also noted that their adolescents spent hours engaged in screen-related activity, and prioritized devices over daily activities such as homework, dressing and eating. Addictive behaviors were particularly prevalent in relation to mobile phones and gaming devices.

And with the phone… he will be glued on it, like the second he gets up he will come in my room and just sit there, and [I say to him], “Okay, you actually haven’t brushed your teeth, you haven’t had breakfast, you haven’t got dressed”.

Parental knowledge of the use of behavioral psychology incorporated into games and social media– and their concerns about that - were also demonstrated:

There’s a lot of immediate gratification for the players, if you get killed you can start a new game easily. Every time you progress in the game, you’re getting rewarded… or you’re moving up levels, whereas in life you’re not getting that immediate feedback so much.

Deceptive behavior was also reported. Parents said that their kids snuck out during the night to get access to confiscated devices or to turn the Wi-Fi back on. They also reported the use of devices in secret in bed at night or in the toilet and even having a secret or hidden device.

There’s just been so many occasions where he’s done things that I would never have even dreamt it was possible, in order to get on [his device]… Because at one stage he was actually getting up in the middle of the night to play games while we’re all asleep.

Other deceptive behaviors included secret and fake social media accounts, switching screens on devices to hide content, and obtaining passwords for accounts and devices without parental knowledge.

And they all have Instagram accounts but then they have private [account] one, private two, private three and you’re friends with mum on one but no way does mum know you’ve got these private ones.

Next, parents expressed concern about the content the adolescents were “addicted” to.

#### Exposure to harmful content: from the ‘inane’ to the ‘inappropriate’

Concerns centered around content that was seen as inappropriate, such as “the amount of pornography they can easily get hold of”. Parents with daughters perceived the impact of social media, cyberbullying, grooming and early sexualization as problematic. “I do worry about all the body image stuff. And all that sort of, objectification and all those kinds of pressures.” In contrast, parents of sons appeared concerned with addictive behavior, gaming, and the viewing of pornography.

There was concern about content that portrayed the world in an overly positive or negative manner, which could in turn lead to young people having unrealistic expectations about the world. Overly positive content set up false or unrealistic presentations of other people’s lives:

Girls… seeing all these images of perfection and things, and they think that’s normal. It’s not… Everyone’s putting their best moments… going on trips and doing this but it’s only their best… and they think that’s real life, sort of thing, that everyone’s always going here and doing this and having a glamorous life.

On the other hand, content that depicted violence or other negative behavior could “lead to a fake sense of danger” and exacerbate anxiety:

I think there’s… a growing sense that our young people are exposed to a lot of information around the negative stuff of life and how bad everything is, and it’s easier for them to fall into this idea, that there’s this alternate reality that’s horrible. Some of it’s true but it’s hard to live in that space for a young person I think and that causes a lot of anxiety.

Whereas concern about pornography and violence might be expected, there was also concern about the banality of content that absorbed adolescents for hours at a time. Participants expressed concern about online content that was perceived as “trivial”, “inane”, “mindless”, “a complete waste of a wonderful mind and body”, and that engaging with such content was “to the detriment of other things that [the young person] could be doing in their life”.

Parents who viewed screen use more positively spoke of adolescents who exhibited less problematic behavior, and could make good use of appropriate, rather than harmful, content:

Overall I’m not worried about it, because he has got a good balance of what he likes to do. And also, the things that he’s watching are helping him. They really have helped him grow and helped him learn.

The only thing we tend to focus on is the amount of time our kids are on it, and the.

damage it’s going to do to their brain. No-one talks about the educational impact, and how that can actually help kids.

The educational impact might, hopefully, “help kids” in the real world, but mostly, parents were concerned about how much their young people were living in a “virtual world”.

#### Living in a virtual world: ‘you can use screens to kind of hide from the world’

Parents spoke about how devices distracted and isolated their adolescents from the “real” world. Spending too much time on screen meant that “you’re not really living life” and there were risks that adolescents isolated themselves or failed to participate in activities. For example, “You can use screens to kind of hide from the world and do it all very much at arm’s length… you kind of shut yourself off.”

For some families, their adolescents no longer engaged in activities that were previously enjoyed. “She used to read all the time, she hardly reads a book now. Or if she does read, it’s on the computer or on her phone.” Or, in some cases, parents saw the young people failing to engage in any activity that did not involve screens. “My son, he would rather, he doesn’t want to go out and play.”

Furthermore, there were some reports of adolescents living “others’ lives” through social media, unable to distance themselves from on-screen narratives about people and events only imagined:

I find it fascinating that we had a group of students who had never met this person who committed suicide, but just knew them through social media and it was like they had lost their best friend.

One of [my son]’s friend’s friend’s friend down the line had an accident on Saturday night and they see it immediately. He wants action immediately, we need to go and help… and then he becomes all anxious and I’m saying, “No, but you don’t actually know the person”.

The virtual world operated at the expense of opportunities for real-life friendships, where screen time was seen by some parents as causing poorer quality social interaction with friends and lack of face-to-face interaction:

At break times, all the boys in Year 9 [ages 13–17 years] would sit in the playground on their iPads playing games and just not talking to each other. And he was still wanting to make friends and interact, and he was finding that that wasn’t very easy.

Parents reported their concern about online “friends” not being friends in real life: “It doesn’t facilitate real friendship and connection– even though they think it does.” This was viewed as a barrier to their adolescents’ ability to develop real-life relationships and interaction skills.

It’s the whole thing about being able to have a real conversation with somebody and understand the facial expressions and the tone of voice… They’re beginning to lose the understanding of that, because they do everything online.

Poor interactions with family were also noted, due to distraction from devices:

Just the fact that they’re just withdrawn from family life… they don’t really even hear you… they realize you’ve said something, are sort of a bit panicked about what they may have missed by floating away.

The nature of online friendships was also of concern. For example, parents saw how inappropriate or negative friendships easily developed online, which could result in bullying or grooming; and there was the issue of not knowing who was at the other end of an online interaction:

Grooming - that’s the thing that really worries me. It’s worried me with all four of [my kids]. Like you could be doing something… totally innocently and then… it’s not a 13-year-old at the other end, it’s a pedophile.

#### Physical, mental, and cognitive harm: ‘when it came to the phone, she hit me in the face’

Concerns over negative outcomes on physical health (e.g., on eyesight, hearing, physical exercise, and sleep), mental health (e.g., anxiety, depression, self-harm and low self-esteem) and cognitive processes (e.g., impaired school performance, reduced attention spans, inability to focus and concentrate) were frequently raised by parents.

A range of physical ill-effects were reported including tiredness, sleep difficulties, back issues and impacts on vision: “[Name of son] just got glasses about two weeks ago and his eyes had been fine up until then”. Hunching over screens in bed led to the young person having “sore eyes, back problem, because… [they] might be lying on the bed, few pillows on their back, hunching and doing that.”

Of more concern to many were the mental and emotional effects, enacted through changes in mood, attitude and behavior, such as irritability, grumpiness and rudeness. “If he’s been on his phone or whatever for a period of time, he’s the most irritable, short-tempered, badly behaved child. So it does, I think, really affect his behavior.” Negative feelings, sometimes building from addict-like behavior and reactions to harmful content, spiraled into emotional outbursts, with anger, fighting, conflict and aggression reported.

There was aggression particularly from parents’ attempts to limit the young person’s screen time via withdrawal of either the device or the internet. “I actually went into the pantry and turned [the Wi-Fi] off and my son got quite aggressive. Not physically, but quite stand-over tactics to me… and I felt that was not on.” Instances of more general fighting and aggression were also described by parents:

I tried to take my phone off my daughter, and I got hit in the face by her and she doesn’t hit me in the face, but when it came to the phone, she hit me in the face.

Aggression between adolescents in the household was also noted, particularly after viewing certain types of content, programs or games:

The way they communicated with each other after they would spend an hour watching those programs was not what I wanted for them. And I could see the tone of voice change, and the attitudes to each other change when they would watch those things, and so I cut it out.

More serious mental-health concerns were also discussed. Parents reported anxiety and depression associated with screen use in their adolescents: “I feel that he can be quite negative and down, depressed, and it’s hard to get him out of that mood. So I do see that.” There was marked social withdrawal, reduced verbal communication, reluctance to spend time with family and a sense of the adolescent as “not actually there”, even though physically present in the home:

He boxes himself in his room, he won’t come down if there are people for dinner, he stays in his room and he won’t come down, he’s very antisocial and this is a huge thing.

Some parents reported that they were aware of instances of suicide and self-harm in other young people. “That seems to impact on their mental health, having just seen that with my own girl.” Links between on-screen and real-world harm were evident; for example, “I don’t think that we would have anything like that problem [of self-harm] that we have at the moment without social media.”

Cognitive functioning was also of concern. Parents reported that their adolescents had a reduced ability to concentrate; for example, “He struggles to sit down and focus for an extended period of time… so studying is difficult.” Reduced effort on schoolwork was described and contrasted with effort put into online gaming: “I think it does reduce their ability to want to learn because if my son put as much effort into his schoolwork as he does into games, he’d be an A in every subject.” Some parents had already seen a negative impact on academic performance, where results went “from 8s and 9s [out of 10] in tests and [being in the] gifted program, to actually pretty much scraping along.”

### Parental management tries to balance adolescent screen use: ***‘for us it’s been not making screens a bad thing’***

Parents were motivated to reduce the amount of time their adolescent spent on screens, protect adolescents from harmful and inappropriate content, and ensure a balance in their children’s lives. A range of strategies were implemented to manage screen use.

Most parents said they had family rules in place; for example, that devices could only be used in certain areas of the house, or at certain times of day. The most common areas banned were the bedrooms and at the dinner table. “We have no devices at the table when we eat and we try and have family dinner every night… [with] a blanket ban on devices at the table.” While restrictions around device use were largely in place for the adolescents, in some families, the same rules applied to all. This was seen as important in terms of accountability and in relation to modelling good habits to their offspring. Time limits were also common (e.g., 30 or 60 min at a time or an overall limit for the week). These strategies were generally perceived to be effective, except for instances where adolescents were displaying signs of addiction or engaging in deceptive behavior. It is also worth noting that this strategy appeared to become less successful and more difficult to manage as adolescents grew older.

Parents also reported the withdrawal of devices, usually as a consequence of extreme use. However, parents noted that the effects of this strategy were limited, and for some families, withdrawal often resulted in significant conflict. “Taking the devices away has only been a couple of times when we’ve had major blowouts and that’s the ultimate punishment, taking away the device.”

Some families used software specifically designed to control internet access, either controlling the Wi-Fi, specific users/devices or the sites and content that could be accessed. Parents who were unaware of such software were interested to learn more about it. Parental access to devices and accounts was another common strategy, especially to personal phones and social media. Parents could view and check browsing histories and social media conversations to ensure nothing inappropriate was taking place. Some parents said they accessed their adolescents’ devices and/or accounts without their knowledge, but for most parents, this access was overt:

I’ll just walk in and demand her device and say, “Hand it over, I want to see”… She knows that the deal is [that can happen] without any cautions, without deleting anything, without throwing a drama, otherwise the privilege is gone.

Honest, open, two-way communication was seen as an important strategy by many parents, with adolescents encouraged to speak to their parents if they had viewed explicit or upsetting content. Conversations included the positives and negatives of screens both in terms of content viewed and device used. In families where this occurred, the strategy was generally reported as being very effective. Understanding screen use from the young person’s perspective was seen as an important element.

I found pornography on his iPad and we had a conversation around that, a very helpful conversation around why it’s not healthy and the problems that are linked to that.

Talking about is, not so much just in terms of what she’s allowed to do or not do… but just trying to have some conversations about the whole kind of thing. Trying to remember to listen to her as well, rather than just lecture her.

For some parents, encouraging their adolescents to develop self-regulation around devices was important. This involved giving clear messages to their adolescents regarding the importance of self-control and responsibility and exercising their own judgement regarding device use and content view. “They need to learn how to restrict themselves and how to balance themselves.” Parents also reported substituting screen use with other activities as an effective strategy to reduce screen use. For example, they would encourage outdoor activities, interacting with friends in person, and participating in family activities. The benefits of reduced screen time were sometimes also acknowledged by the young people themselves, who could see the positive effects:

And [my daughter] even recognized that… she said, “It must be what it’s like for people who come off drugs, it’s almost like you feel like an addiction to it, even though you think you’re in control of it” and she said, “When you actually do turn it off, you realize that actually, you do start to connect more with people.”

Parents were specifically asked whether screens were used as a punishment or reward. In general, screens were not used as a reward but the removal of a device or internet access were used as a punishment by some parents. However, some families were against the removal of screens. “I think for us it’s been not making screens a bad thing… not withdrawing them [screens] as a punishment.” There was also an indication that using screens as a reward or punishment worked better for younger children and was less effective with adolescents.

### Barriers to managing adolescent screen use are manifold: ***‘no-one wants to be mean to their kids, right?’***

Although parents had attempted various strategies to reduce adolescent screen time, they noted numerous barriers to managing screen use. Some parents noted that it was easier to let the screen use continue rather than enforcing restrictions and potentially causing conflict. Associated with this was a strong sense from parents that it was important to “pick your battles” and that “sometimes it’s easier just to leave it than make an issue out of it”.

Parents spoke about the difficulties in following through on some of the family rules and strategies, as they would get “busy” or “distracted”.

“Okay you’ve got an hour of devices” and then we forget to check back in again because we’re running around. And then we’ll put these new strategies in place, and then we forgot that we’ve put this plan into place four days later.

Parental screen use was seen as a particularly tricky issue for parents who wanted to be seen as modelling good behavior.

I think there’s a lot that we have to do with modelling, and it’s good she sometimes does call us on it like, “You sat there on your phone, Mum, you’re meant to be watching [a movie] with me”.

A common barrier to management for parents was the need for adolescents to have their devices for other purposes (e.g., laptops for homework, phones for safety/staying in touch).

They do everything on Google Docs… you’re told they need a device. So therefore, you can’t use the “I’m just going to take the screens away” [response]. It’s a big impediment.

Some parents also spoke about the lack of technical knowledge that made implementing strategies difficult. Parents noted that their adolescents had greater technical knowledge than did they, which meant that the young people were able to circumvent any controls put in place. “The last sort of software I put on like that, it was deleted within an hour…by him… They know more than we do.” Deceptive behavior, discussed above, was also a barrier for parents to implement successful strategies. For example, a parent may insist on all devices being kept in the communal living area overnight, but the adolescent could sneak out and take it back once the household was asleep.

A small number of parents expressed concern that limiting device use may result in social isolation as “their devices are their connection to their friends”. Other barriers included parents not being on the “same page” as each other (particularly in split families), the fact that having set times for the Wi-Fi to be turned off did not suit irregular schedules, and that for young people already experiencing mental health issues, withdrawal from their device could cause further anxiety. There was also some concern that, if device use was restricted, it could affect the young person’s future opportunities by limiting their access to educational content and information or preventing them learning the technological skills that would be needed in the future. Lastly, adolescents often used guilt as a strategy to get parents to back down, insisting that their parents were stricter than other parents:

Well, I guess the other thing is they constantly say, “You’re tougher than other parents”… that’s how the children wear parents down, because they don’t want to be mean. No-one wants to be mean to their kids, right?

### We think a range of strategies could help: ***develop a toolbox***

Participants were asked to consider what they would find helpful in an intervention that might be designed to assist parents with managing adolescents’ screen use. There were ideas for the content and strategies that could be taught, and the need for both schools and the young people to be involved in developing any interventions. A popular suggestion was of parents being provided with a ‘toolbox’ of information and strategies. Managing screen use was not a one-size-fits-all scenario, “You need to have a range of strategies to pick and choose and if one doesn’t work [have] something else to try.” Table 2 presents key toolbox items and comments.

*Parenting style.* Alongside a toolbox of parenting strategies relating to screens, in particular, there was also an acknowledgement by some that the management of the issue was very much related to general parenting skills, rather than technology per se:

I think this issue has got a lot to do with parenting. So when I was growing up it was about the TV, and my dad was always onto me watching TV all the time. So I think it’s around how we parent, I think the technology’s shifted. The biggest problem I find with our parents at our school, is they just say, “Oh when I say to my kids, oh they just won’t listen”. Well, that to me is a parenting issue, not a technology issue… I think we almost have an issue just in the general parenting, which is why people blame technology, but it’s actually parenting in general.

## Discussion

Parents provided rich accounts of the concerns and challenges of adolescent screen use in their families and communities, and practical ideas for resourcing more skillful responses to young people grappling with an addictive digital existence. Screens were a part of adolescents’ daily life, yet there was a sense of parents being caught off-guard as they dealt with the first generation of adolescents who have never known life without the worldwide web in their pocket.

Participants’ deep concerns centered on both the risks they were aware of, plus the actual behavior witnessed, in terms of (1) addiction, (2) harmful content, (3) the dominance of the virtual over the ‘real’ world and (4) worrying impacts on physical, mental and cognitive wellbeing. Defining and measuring “addiction” in the context of social media use is debated [27], but the evidence is increasingly of concern. Participants’ comments about the “addiction” evident in their adolescents echo more recent online survey research with parent/child dyads (surveyed separately) during the COVID-19 pandemic (mean age 11 years) [28]. The intensified screen use during the pandemic highlighted that it was not the frequency of use, but the addictive quality that seemed to be linked to adolescents’ emotional, behavioral and academic difficulties; namely, where the screen was used to try to manage behavioral and emotional issues, in the face of pre-existing vulnerabilities and/or authoritarian or mismatched parenting styles [28]. The adolescents could spend hours online and be relatively OK in the context of a “positive” parenting style (showing support, warmth, and positive reinforcement), endorsing the evidence that available and supportive parenting is a source of resilience, including in times of crisis [29]. This accords with our participants’ recommendations that the quality and engagement of the parent-adolescent relationship is key, and that help via a ‘toolbox’ would support building skills to achieve that.

Our participants’ struggles with managing adolescent screen use and working together to find ways to move between the digital and real worlds highlight that parenting alone—however “positive”—is insufficient. The power of the feedback and rewards, that parents mentioned as designed by behavioral psychologists, drive screen-users into addiction and the virtual world [30], and more understanding of this needs to go into a parental ‘toolbox’, as they suggest. Recent research shows that high amounts of screen activity result in structural patterns of change in the brain that mimic changes seen in those who start drinking at a young age [31]. 

Other key concerns—exposure to harmful content and the risks of living in a virtual world—were vividly described by our parents, in a way that more quantitative calculations of hours on-screen or underage access to ubiquitous porn sites perhaps do not. Debates persist as to whether concern about the harms of screen use are a form of “moral panic” [10, 15]—participants were aware of their own parents’ concerns about television, rock music, violent computer games and so forth, back in their day. Since our data were gathered, the global pandemic has by no means settled these debates, with the combination of real-world catastrophic features (death of loved ones, large-scale illness, indeterminate lockdowns, isolation and school closure), and technological power (vaccines, epidemiological and public health advances, online communities and education) poised in tension, alongside diverse responses to pandemic life [32]. The narrative in the media now is tending to be all about how tech can be good and bad, implying a balance. However, the evidence—whether from our study or the US Surgeon General’s review [9]—increasingly supports the idea that the harms seem to outweigh the positives. We need to change the narrative around adolescents and screen use [33]. 

This is particularly in relation to adolescent mental health, an aspect that our participants were concerned about in relation to screen use, both from what they had read or heard (e.g., about suicide and self-harm in other young people), but also from what they saw with their own adolescent being “down, depressed”. Research debates continue as to the effects of social media use on adolescent mental health, with some arguing that there may be negligible affects overall for many young people [34–36] and caution needed in interpreting aggregate-level statistics and effect sizes in relation to individual adolescents, some of whose wellbeing will be negatively affected by social media use while others are less affected [37]. However, many of these studies do not account for relative risk [33] and rely on self-report of social media use– which can be inaccurate. As Panayiotou et al. (2023) note, “a limitation of the social media literature that we are unable to address [is that] our variable of social media use reflects duration (time spent on social media) as estimated by the individual” (p. 318). A recent quasi-experimental study is one of the first to demonstrate a causal link between social media and youth mental health issues [38]. In this study, the mental health of students on US campuses was measured during a staggered introduction of Facebook, and it was found that the introduction of Facebook resulted in significant increases in the likelihood of reporting symptoms of depression and anxiety [38]. Also, recent brain studies [31] show that not only does screen use structurally change brains, but this change (somewhat) explains the relationship between screen use and anxiety. More qualitative research, where the detailed circumstances of parents and young people in relation to mental health issues is needed [20]. Mood changes, aggression, social withdrawal or chaos, emotional lability and boundary testing (including deceptive behavior) can also be part of adolescent social and emotional development [8], hence our participants’ wish for toolbox explanations of developmental stages and “what is going on in this teenage brain”.

Participants’ concerns about the physical health effects of screen use included eye strain (e.g., needing glasses); research during the intensified use of screens for education in the COVID-19 pandemic bear this out, with risks of higher rates of eye strain and myopia in some young people reported and strategies to mitigate these suggested [39, 40]. The “hunching”, back problems, sleep problems and tiredness noted by our participants became more prevalent across adult screen use in the pandemic, too; the information toolbox that they recommend should include the latest tips on managing these risks for us all. The cognitive concerns about an inability to focus on schoolwork in the face of screen appeal were tested by the pandemic, during periods when the only schoolwork available was online.

There are calls for improved parental awareness to better manage adolescent screen use, alongside acknowledgement of the challenges of doing so [41]. Our participants were aware of the role-modelling influence of their own screen-use habits on their adolescent children, who called them out on it, as other research notes [16, 39]. Awareness of “over monitoring” by parents is also needed [42] and our participants were aware of needing to ‘pick their battles’; however, parents do not usually let their kids drink because it is too much effort to stop them; maybe screen addiction is a ‘battle’ worth fighting. An interview study with adolescents and parents in Singapore echoed the difficulty and frustration with both implementing rules and monitoring adolescent’s compliance with them around screen use that our participants felt [43]. Furthermore, some research points to the crucial need for social and cultural values to inform and strengthen parenting strategies [44, 45]. Our participants wanted “local” (vs. “American”) information, research and strategies in the toolbox, and future research should underpin this. More nuanced exploration of cultures, gender, social positioning and parenting styles would cast further light on practical tools for our parents; for example, a US qualitative study during the pandemic highlighted the gendered support for adolescents (in favor of boys) around technology that was of concern [46]. 

*Limitations.* Data were collected, analyzed and written up, ready to flow into the next project stage of developing an intervention to pilot with parents, when the COVID-19 pandemic arrived. Aotearoa New Zealand went into nationwide lockdown in March 2020 and the use of screens increased exponentially for adolescents’ socially distanced school and social life across the globe. Yet, 3 years on, “in-person” life is being reclaimed—including by adolescents themselves in education and social life [47]—alongside expanded screen use, and we consider our participants’ views are still vital to be heard. Ideally, if there was funding, we would return to them; we suspect their concerns about addiction and the physical, mental, cognitive and emotional costs of screen management would only have intensified, but also new ways of working with screen use would have had to develop. Even better would be to interview their post-pandemic adolescents—would they still “hit [a parent] in the face” in defense of a phone? Future research incorporating adolescents’ voices would enhance understanding of the family dynamics around screen use. Also, we recruited parents in our study who were already concerned about adolescent screen use (and they remain in the majority in Aotearoa New Zealand [48]); how would the minority of parents not identifying themselves as “concerned” reflect on device use by their adolescents? Are there different parenting skills or family dynamics operating there? Additionally, there can be a disconnection between how parents feel about screens and their wish or ability to articulate those feelings in an interview/focus group format [49]– culturally diverse research approaches with both adolescents and parents would be enlightening.

## Conclusion

This study makes a unique contribution to a global conversation before and after a pandemic. The parents provided rich insights about responding to adolescent screen use from their concerned perspective; data analysis distilled key areas of trouble and practical ‘toolbox’ interventions to develop. A global pandemic struck and now we are presenting our participants’ ideas, in the light of recent literature that is trying to make sense of adolescent screen use and parenting out the other side of that pandemic. The scale has changed, and the challenge of parents ‘picking battles’ is at risk of becoming ‘losing battles’, as they try to care for their offspring in the face of powerful marketing and denial, and increasing evidence of harmful screen use, making their practical toolbox ideas even more important to enact.

**Table 2:** Navigating adolescent screen use: a toolbox for parents

Toolbox items	Participant comments
**1. Access to sources of practical, local scientific information**	• The things that would interest me most would be academic research on [screen use] because that’s where I think they get very balanced arguments. It’s good to have New Zealand research or Aussie research, and not the American research… so we’re talking about us here in the Pacific. • I think some basics about scientific information about screen use, and the effect it has on families and children’s development… the long-term effects, what’s happening with these children.
**2. Examples of techniques that have worked for other families**	• Maybe real-life interviews on what’s worked for other families… YouTube things on discussions on families and things. Discussions on modelling behavior for the parents just to be mindful of what they’re doing.
**3. Teaching parents how to teach life skills for managing screen use, e.g., prioritization, mindfulness, self-regulation of behavior**	• Would be really good if there were ways that you could manage screen time but in partnership with your child. • Start early, start early somewhere, well before they become teens… The horse has already bolted for us, it’s more about pre-teens or whatever.
**4. Strategies specific to a child’s age and/or personality**	• [For example] if you had a moody teen you could try this, if you had a teen who was the oldest, this might work because we know older children react like this to certain things… without putting everyone in a box. • Get a psychologist or a teen behavioralist to explain to parents what is going on in this teenage brain because sometimes it doesn’t matter what we do, we can’t change that wiring in their brain and they’re going to have to work through the normal developmental stages to come out the other end.
**5. Techniques for having productive conversations with adolescents**	• Having a good conversation about it that doesn’t quite quickly result in heightened emotions and [the young person] getting super defensive and feeling attacked, and me getting grumpy… Tools for having some good conversations around that.
**6. Ideas for beginning conversations about social media, inappropriate content and addiction**	• Being able to have those conversations… like the key building blocks when they start to get to that age of wanting to access social media and stuff like that, so maybe having guidance around those conversations, conversation starters even, and points to cover, that would be helpful. • And also recognizing signs where kids are getting too addicted… if people have experiences with that kind of thing, whether that might be a sign that their kid’s playing far too much, or behavioral changes, behavioral signs, that kind of stuff.
**7. Tools to help engage the whole family in relation to devices**	• Something that you can actually do to use it as a positive family thing as opposed to just something that an individual goes and does by themselves.
**8. How to work with boundary-setting**	• How to set boundaries or “rules and regulations”, how to monitor and maintain them… how to deliver in a way that is non-combative. • Tips and tricks, just to be aware these are the things that teenagers are doing to bypass your systems or this is how they do things incognito… The kinds of apps that are available if you have to monitor that.
**9. How to manage those who are displaying problematic behaviors such as addiction**	• Recognizing it in kids that are more addicted to games than others and how to work with those kids is probably a huge thing… it’s understanding what makes those kids get so hooked on these games and how to stop that.
**10. The importance of different parenting styles in relation to screen use**	• If you’ve got an “authoritarian” parenting style or an “authoritative” parenting style, that can determine a lot on how things work at home… if you can shift your parenting towards authoritative, where there’s high expectation but also high connection, you’re going to get better outcomes from that.
**11. Build a non-blaming community intervention**: ***Build courage in each other***	• You don’t want parents going away feeling like, “Oh I’ve stuffed up because I’ve done this and we let them do that.” It’s got to be really positive and affirming of what can be done, and with a hopeful outlook of how technology can be helpful, but how to keep it in its right place. • Parents’ meeting (face-to-face and online) would focus on the importance of “knowing you’re not alone” and “feeling like you’re in this together”; a potential opportunity for “building courage in each other”.
**12. Work with young people**: ***There’s always two sides***	• So I was imagining like a whole family going along and doing a workshop rather than just the parents and then coming home and delivering the bad news that actually we’re restricting everything. • Have a “teen panel” at the intervention, to try and get both parents and adolescents “on the same page”.
**13. Work with schools**: ***Build digital citizenship***	• I think the children at school get quite a fair amount of training on digital citizenship. But it would be nice for us to actually also see what they are being told… can you actually do that for the parents as well, like we can see it. • Awareness of policies about device use, homework etc. - if they’re using their device, it’s very easy for kids just to say, “It’s schoolwork, it’s schoolwork.”

## Data Availability

The qualitative data used during the current study are available from the corresponding author on reasonable request.
